# Classical and Paradoxical Effects of TNF-α on Bone Homeostasis

**DOI:** 10.3389/fimmu.2014.00048

**Published:** 2014-02-13

**Authors:** Bilal Osta, Giulia Benedetti, Pierre Miossec

**Affiliations:** ^1^Immunogenomics and Inflammation Research Unit EA 4130, Department of Immunology and Rheumatology, Hospital Edouard Herriot, University of Lyon 1, Lyon, France

**Keywords:** TNF-α, bone homeostasis, osteoblast, osteoclast, rheumatoid arthritis, ankylosing spondylitis, osteoporosis

## Abstract

Tumor necrosis factor-α (TNF-α) plays an essential role in the regulation of bone homeostasis in several chronic immune and inflammatory joint diseases, where inhibition of TNF has led to significant clinical improvement. However, TNF-activated pathways and mechanisms involved in bone remodeling remain unclear. So far, TNF-α was known as an inhibitor of osteoblast differentiation and an activator of osteoclastogenesis. Recent contradictory findings indicated that TNF-α can also activate osteoblastogenesis. The paradoxical role of TNF-α in bone homeostasis seems to depend on the concentration and the differentiation state of the cell type used as well as on the exposure time. This review aims to summarize the recent contradictory findings on the regulation of bone homeostasis by TNF-α at the isolated cell, whole bone, and whole body levels. In addition, the involvement of TNF-α in the bone remodeling imbalance is observed in inflammatory joint diseases including rheumatoid arthritis and ankylosing spondylitis, which are associated with bone destruction and ectopic calcified matrix formation, respectively. Both diseases are associated with systemic/vertebral osteoporosis.

## Introduction

Tumor necrosis factor-α (TNF-α) is one of the several pro-inflammatory cytokines involved in bone remodeling. Classically, TNF-α plays a major role in the regulation of bone homeostasis by stimulating osteoclastogenesis and inhibiting osteoblast (OB) function. It is also involved in the pathogenesis of chronic inflammation in both mouse and human models. Based on these observations, treatments of several chronic inflammatory diseases such as Crohn’s disease, ulcerative colitis, and psoriasis target TNF-α. TNF-α is also associated with the pathogenesis of inflammatory joint diseases including rheumatoid arthritis (RA), which is characterized by massive juxta-articular bone destruction, and ankylosing spondylitis (AS), which is characterized by simultaneous bone destruction and excessive formation, respectively. However, opposite findings suggest that TNF-α may also induce osteogenic differentiation. This review summarizes the classical and paradoxical effects of TNF-α on bone homeostasis by focusing on osteoblastogenesis and osteoclastogenesis.

## TNF-α and Its Signaling Pathways

The immune response is modulated by several inflammatory cytokines including TNF-α. TNF-α acts through several pathways including the activation of nuclear factor kappa-B (NF-κB) involved in inflammation and apoptosis (1). TNF-α is produced mainly by monocytes, but can also be secreted by many other cell types (1–3). It is produced as a transmembrane protein (mTNF-α) that is subsequently cleaved by the TNF-α converting enzyme (TACE) to a soluble form (sTNF-α) (4). Both types are biologically active and bind as a trimer to either TNF receptors TNFR1 (also known as TNFRSF1A or p55) or to TNFR2 (also known as TNFRSF1B or p75).

Activation of TNFR1 death domain initiates the apoptotic signaling through the activation of several factors such as caspase and NF-κB resulting in transcription of pro-inflammatory cytokines, chemokines, and anti-apoptotic molecules (5). Unlike TNFR1, the cell expression of TNFR2 is far more limited and does not contain a death domain. Its effects include T cell activation and proliferation via signaling pathways that involve NF-κB, activator protein 1 (AP-1), and the mitogen-activated protein kinases (MAPKs) (6). The pro-inflammatory effects of this cytokine are well documented (7) and are involved in many osteo-immunological diseases including RA and AS (8).

In humans, TNF-α antagonists are clearly effective in a large number of conditions, as first shown in patients with RA (9, 10). However, incidents of lupus-like syndrome and skin lesions have been reported in patients receiving anti-TNF therapies (11). The cellular and molecular mechanisms underlying the TNF-α-mediated pro-inflammatory effects and the occasionally paradoxical effects of TNF-α antagonists in chronic inflammatory diseases remain incompletely understood (10). This also applies to bone biology.

## Bone Biology

Bone structure is affected by several paracrine and endocrine factors including mechanical abrasion and serum electrolyte levels such as calcium and phosphorus. This remodeling is regulated by several cells such as osteoclasts (OCs), OBs, and osteocytes (OYs) (12). This continuous remodeling prevents and removes fatigue-related micro-damage and allows adaptation of the bone mass and structure. The number and activity of OCs and OBs are determined by a multitude of factors such as hormones, cytokines, and locally produced signaling molecules under the influence of mechanical stimuli (13–15). Among these factors, TNF-α was found to have great effects on both bone regulator cells, OBs and OCs.

### Biology of osteoclasts

**Figure 1:**
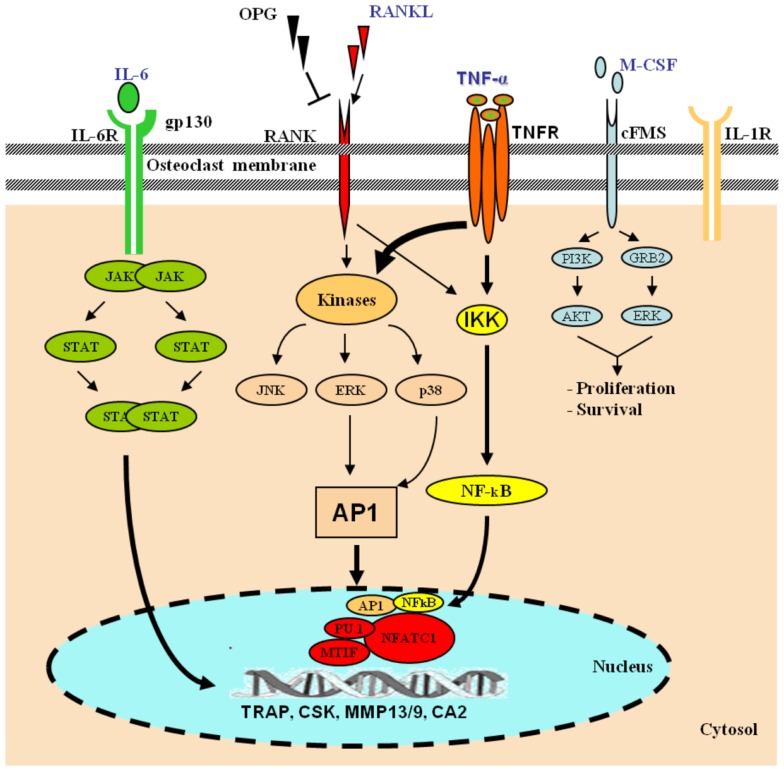
**Role of TNF-α in osteoclast differentiation**. Hematopoietic stem cells differentiate along the myelo-monocytic pathway with M-CSF stimulation. Differentiating cells continue along a trajectory toward the osteoclast phenotype under the influence of RANKL, which signals through the transcription factors AP-1 and NF-κB. In the presence of RANKL, various pro-inflammatory cytokines, including TNF-α, IL-6, and IL-1, cooperatively orchestrate enhanced osteoclastogenesis. TNF-α signals via NF-κB and the mitogen-activated protein kinases (MAPKs), and IL-6 via the JAK–STAT pathway. The activation of osteoclasts with the inflammatory mechanisms mentioned leads to exaggerated systemic and local bone loss. [AKT, protein kinase B; CA2, carbonic anhydrase 2; CSK, cathepsin K; ERK, extracellular signal-regulated kinase; IKK, IκB kinase; JAK, janus kinase; gp130, glycoprotein 130; GRB2, growth factor receptor-bound protein 2; MITF, microphthalmia-associated transcription factor; MMP, matrix metalloproteinase; NFATc1, nuclear factor of activated T-cells, cytoplasmic 1; PI3K, phosphoinositide 3-kinase; SPI1, spleen focus forming virus (SFFV) proviral integration oncogene spi1; STAT, signal transducer and activator of transcription; TRAP, tartrate-resistant acid phosphatase].

Osteoclasts are hematopoietic cells derived from the monocyte lineage. They undergo a series of differentiation steps to become mature bone-resorbing cells. Intracellular signaling cascade of transcriptional regulation of osteoclast-specific genes is activated by macrophage-colony-stimulating factor (M-CSF) and receptor activator of nuclear factor kappa-B ligand (RANKL) (16) (Figure 1). Upon differentiation, OC precursor cells express strongly RANK, the receptor for RANKL.

### Biology of osteoblasts

**Figure 2:**
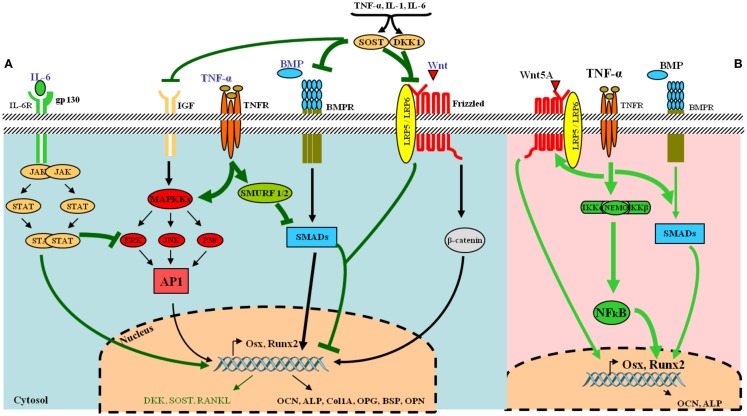
**Tumor necrosis factor-α and its osteoblastogenesis paradoxical effects**. **(A)** Under normal condition, various receptors and downstream signaling pathways can be activated in osteoblasts. The most common ligands, receptors, and their signal transduction are represented in black. Essential pathways involve bone morphogenetic proteins (BMPs) and their receptors, acting via SMAD proteins to directly or transcriptionally activate RUNX2 and its subsequent downstream cellular events and the Wnt–frizzled pathway, which utilizes β-catenin for further activities. Under inflammatory conditions, the release of pro-inflammatory cytokines such as TNF-α and IL-6 leads to the inhibition of osteogenic differentiation via several mechanisms (represented in green in the figure). IL-6 inhibits mitogen-activated protein kinase (MAPK) activities by activated signal transducers and activators of transcription (STAT). TNF-α activates SMAD ubiquitination regulatory factor-1 (SMURF1) and SMURF2 leading to the inhibition of SMADs. The pro-inflammatory cytokines also up-regulate dickkopf-related protein 1 (DKK-1) and sclerostin (SOST), which inhibit the Wnt–frizzled pathway, whereas many other osteoblast gene products are down-regulated. **(B)** TNF-α can also favor osteogenic differentiation via NF-κB by increasing expression of BMP-2, Osx, Runx2, OCN, and Wnt pathway. [AP-1, activator protein 1; BMPR, BMP receptor; ERK, extracellular signal-regulated kinase; FGF, fibroblast growth factor; gp130, glycoprotein 130; IL-6R, IL-6 receptor; JAK, janus kinase; JNK, JUN N-terminal kinase; LRP5-6, low-density lipoprotein receptor-related protein 5 or 6; Osx, osterix (Sp7); OPG, osteoprotegerin; ALP, alkaline phosphatase; BSP, bone sialoprotein; OPN, osteopontin; OCN, osteocalcin; p38, p38 MAPK].

Osteoblasts are derived from pluripotent stem cells called “mesenchymal stem cells (MSCs)” capable of differentiating into adipocytes, chondrocytes, fibroblasts, skeletal muscle cells, and tendon cells (17). A number of factors can stimulate the differentiation toward an osteoblastic direction. The first step includes the proliferation of osteoprogenitors leading to the production of proteins such as histones, fibronectin, and type I collagen. Subsequently, osteogenic matrix is formed through the expression of genes responsible for the production of alkaline phosphatase (ALP), bone sialoprotein, and type I collagen. Finally, extracellular matrix is mineralized by activation of genes involved in the release of osteocalcin (OCN), osteopontin, and collagenase. This complex cascade of gene expression and cellular differentiation is orchestrated by several transcription factors including the runt-related transcription factor-2 (Runx2) and surface expression of RANKL (Figure 2A).

### Biology of osteocytes

Osteocytes are derived from MSCs through OB differentiation. They are located within the bone matrix and represent 90–95% of all bone cells in adults (18). In addition, they have the longest life span among bone cells, up to decades, within their mineralized environment (19). OYs are involved in the dynamics of bone structure through expression of different markers that are released during OB-to-OY ontogeny. In addition, OYs affect the function of other organs such as phosphate transport in kidney (20). Furthermore, OYs are now known to be the principal sensors for mechanical loading of bone. They translate the canalicular flow resulting from bone loading into OCs and OBs recruiting signals (21). Due to the high expression of RANKL by OYs, they have a more significant contribution to osteoclastogenesis as compared to OBs and bone MSCs (22, 23).

## Effects of TNF-α on Isolated Cells

### Effects of TNF-α on osteoblasts

Previous *in vitro* studies have indicated an inhibitory effect of TNF-α on OB differentiation (Table 1). This blockage of osteogenic differentiation resulted from the inhibition of insulin-like growth factor-1 (IGF-1), osterix (Osx also known as SP7), and Runx2 (24–26). The inhibition of Runx2 seemed to be mediated by the TNF-induced up-regulation of Smurf1, a negative regulator of OB differentiation that causes the degradation of Runx2 (27). Moreover, TNF-α inhibited MSC differentiation into OBs via the ubiquitin protein ligase Wwp1 in TNF transgenic mice expressing human TNF-α (28). Another human study demonstrated an inhibitory effect of TNF-α on Runx2 and collagen expression. However, an increase in alkaline phosphatase activity and matrix mineralization was noted (29).

**Table 1:** **Overview of studies indicating a TNF-mediated inhibition of osteogenic differentiation**.

Model	TNF-α	Effects	Reference
Osteoblastic cell line and MC3T3-E1 cells	Dose-dependent IC50 = 0.6 ng/ml	TNF-α inhibits osteoblast differentiation via the inhibition of insulin-like growth factor I (IGF-I) expression	(25)
Fetal calvaria precursor cells and MC3T3-E1 (pre-OBs cell)	0.6 ng/ml	TNF-α inhibits the expression of Runx2	(26)
Mice primary stromal cells	1–20 ng/ml	TNF-α inhibits pre-osteoblast differentiation through TNFR1 and by inhibiting Runx2	(24)
MSCs of mice	10 ng/ml	TNFR1 mediates TNF inhibition of OBs differentiation independently of apoptosis	(30)
TNF transgenic mice, C2Cl12 cells, and 2T3 OBs precursor cells	2.5–7.5 ng/ml	TNF-α promotes Runx2 degradation through up-regulation of Smurf1 and Smurf2 in osteoblasts	(31)
Mouse myoblast cell line (C2C12)	10–100 ng/ml	TNF-α inhibits BMP-induced osteoblast differentiation through activating SAPK/JNK signaling	(32)
MC3T3-E1-clone 14 cells	10 ng/ml	TNF-α represses BMP signaling by interfering with the DNA binding of SMADs through the activation of NF-κB	(33)
hMSCs	0.1–1 ng/ml	TNF-α inhibits Runx2 and collagen expression but increases ALP activity and mineralization	(29)
C2C12 and calvaria cells	10 ng/ml	Msx2 mediates the inhibitory action of TNF-α on osteoblast differentiation via suppressing BMP-2	(34)
MSC from TNF transgenic mice	0.5 μg/injection	TNF-α inhibits MSC differentiation into OBs via the ubiquitin E3 ligase Wwp1	(28)
MC3T3-E1-clone 14 cells and C3H10T1/2 cells	10 ng/ml	TNF-α inhibits differentiation of OB progenitors via the homeobox protein Prx1, which inhibits Osx and Runx2 transcription	(35)
C2C12 and primary mouse calvaria cells	10 ng/ml	TNF-α enhances the transcription of Smurf1 in an AP-1- and Runx2-dependent manner leading to inhibition of OBs differentiation	(36)
Mouse ESC (osteo-mESC) and primary OBs cells	–	The pro-inflammatory cytokines IL-1β, TNF-α, and IFN-γ only reduce the formation of bone nodules by primary OBs, and not by osteo-mESCs	(37)

In full contrast with these findings, other murine and human MSC studies have reported that TNF-α can also induce osteogenic differentiation (Table 2). In three *in vitro* rodent models, low concentrations of TNF-α increased osteogenic differentiation via an up-regulation of Runx2, Osx, OCN, and ALP levels (38, 39). Moreover, four human experimental models indicated a similar osteogenic activity for TNF-α. In these models, osteogenic differentiation was promoted via induction of BMP-2, Osx, Runx2, and OCN (40–43). In addition, human models revealed that the dual role of TNF-α on osteogenic differentiation was directly dependent on the concentration of TNF-α, the cell type, and the exposure time (Table 2). Furthermore, a recent study showed that TNF-α stimulated the expression of Wnt5a, which was directly associated with an increase in tissue non-specific alkaline phosphatase (TNAP) levels and mineralization. This suggests an autocrine stimulation of OB activity by Wnt5a in response to TNF in hMSCs (44). This paradoxical effect of TNF-α in inhibiting or activating osteoblastogenesis, lies in the differentiation stage of the responding cells. MSCs can differentiate into several lineages. At this early stage, TNF-α binds its receptors and favors osteogenic differentiation through activation of multiple signaling pathways, notably NF-kB (40) (Figure 2B) (Table 2). On the other hand, TNF-α inhibits osteoblastogenesis in the presence of pre-OB cells, which are already on the differentiation process. This inhibition occurs at different levels, where TNF-α induces DKK-1 expression that inhibits Wnt pathway (45), or activates Smurf that inhibits BMP-2 pathway (31) (Figure 2A) (Table 1).

**Table 2:** **Overview of studies indicating a TNF-mediated activation of osteogenic differentiation**.

Model	TNF-α	Effects	Reference
Human mesenchymal stem cells	20 ng/ml	TNF-α promotes osteogenic differentiation of hMSCs by triggering the NF-κB signaling pathway, which in turn induces BMP-2 up-regulation resulting in the enhancement of Runx2 and Osx expression	(40)
Rat mesenchymal stem cells	5–50 ng/ml	TNF-α stimulates only at high dose the osteogenic differentiation of MSCs grown on biodegradable polymeric microfiber scaffolds. However low TNF dose does the opposite effect	(46)
Human adipose tissue-derived mesenchymal stem cells	10 ng/ml	NF-κB activation by TNF-α stimulates osteogenic differentiation of hADSCs by increasing the expression of the phospholipids–lysophospholipid transacylase TAZ	(43)
Murine mesenchymal stem cell line ST2	0.01–0.1 ng/ml	TNF-α induces osteogenic differentiation via induction of Runx2 and Osx	(38)
Murine models	1 ng/ml	TNF-α promotes fracture repair by augmenting the recruitment and differentiation of muscle-derived stromal cells	(39)
Adipose tissue-derived mesenchymal stem cells (ASCs)	1 ng/ml	In a co-culture system of human OBs with ASCs, HOBs pre-treated with TNF-α for 24 h induced a significantly greater osteogenic differentiation of ASCs than with the HOBs without TNF-α treatment	(42)
Human adipose derived stromal cells	10 ng/ml	Recombinant human TNF-α promotes the *in vitro* human adipose derived stromal cells transformation into osteoblasts via inducing Runx2 and Osx expression	(41)
Human mesenchymal stem cells	1 ng/ml	TNF-α increases the levels of Wnt5a, which in turn stimulates tissue-non-specific alkaline phosphatase (TNAP) levels in an autocrine manner and increases mineralization	(44)
Human dental pulp stem cells (DPSCs)	10 ng/ml	TNF-α triggers osteogenic differentiation of human dental pulp stem cells via the NF-κB signaling pathway	(47)

### Effects of TNF-α on osteocytes

Limited results are available on the effect of TNF-α on OYs, which are known to be affected by the surrounding environment. TNF-α and interleukin-1 (IL-1), which increase with estrogen deficiency, can induce OYs apoptosis (48). In a fluid shear stress study, in which the mechanical bone loading was mimicked by applying pulsating fluid flow, the TNF-α-induced apoptosis observed in OYs was inhibited by mechanical loading. However, this effect was not observed in OBs and periosteal fibroblasts. Since apoptotic OYs attract OCs, these results suggest a key role for OYs apoptosis in osteoclastic bone resorption during bone remodeling that is in part modulated by TNF-α (49). Additionally, TNF-α was shown to inhibit the increase in nitric oxide (NO) production and intracellular calcium while strongly reducing F-actin content. This resulted in a reduction of OYs stiffness and provided a possible mechanism to explain the contribution of inflammation to loss of bone mass (50).

### Effects of TNF-α on osteoclasts

The role of TNF-α as a stimulator of osteoclastogenesis is well established (51–55). The expression of a number of transcription factors, including NF-κB, is critical for osteoclastogenesis (56, 57) (Figure 1). Early bone marrow progenitors embark on a path toward pre-OCs or monocytes–macrophages under the influence of M-CSF while TNF-α, IL-1, and RANKL promote a progression toward the functional OCs phenotype. Thus, both RANKL and TNF-α are not only required for, but also synergize to induce osteoclastogenesis. The requirement for both TNF-α and RANKL allows for a more precise control of OCs numbers through a dual level of regulation. First, they affect the selective signaling of the precursor cells via the two different receptors, and subsequently their synergy further increases the activation of NF-κB and AP-1 signaling. Furthermore, the effect of the pro-inflammatory cytokines TNF-α, IL-17, IL-6, IL-1β, and IL-23 on osteoclastogenesis *in vitro* demonstrated that they have specific characteristic osteoclastogenic properties. However, they exhibited osteoclastogenesis-related activity only in the presence of permissive levels of RANKL. TNF-α appears to have osteoclastogenic properties in the early stage of osteoclastogenesis when bone marrow-derived macrophages (BMMs) are at the stage of osteoclast precursor cells (58).

## Effects of TNF-α on Whole Bone

These results on isolated cells need to be expanded to whole bone studies in order to reproduce the cell–cell interactions between bone specific cells and immune cells in the bone marrow. Only a few studies have examined the effects of TNF alone or combined to other cytokines such as IL-1 and IL-17 in *ex vivo* models or explants where both osteoclast and OB cells are present. In a RA *ex vivo* model of bone resorption, an IL-4-mediated inhibition of TNF-α levels corroborated with an inhibition in bone resorption, manifested as a 35% increase in the mean total bone area with IL-4 (59). Another study with RA-derived bone explants showed that inhibition of TNF-α decreased inflammation as measured by levels of IL-6 and bone resorption markers. These inhibitory effects were enhanced in combination with IL-1 and IL-17 blockade (60). Histomorphometric analysis of bone samples in patients with different diseases affecting bone such as RA, Crohn’s disease, and bronchial asthma showed that eroded surface over bone surface (ES/BS), a parameter of bone resorption, was significantly increased in the context of high TNF-α levels (61). On the contrary, one study showed that addition of TNF-α to a cultured fetal mouse long bone explant induced NO production resulting in suppression of osteoclast bone resorption (62). These findings suggest a mixed effect of TNF-α on bone structure, which corroborates with the paradoxical role of TNF-α observed *in vitro*.

## Effects of TNF-α *in vivo*

Tumor necrosis factor-α disrupts bone homeostasis by activating osteoclastic resorption, inhibiting osteoblastic proliferation and matrix synthesis, and activating TNF receptor-associated Factor-2 (TRAF-2). The latter activates NF-κB, AP-1, and MAPKs signaling pathways, which leads to decreased bone formation.

In human TNF transgenic mice, TNF-α induced DKK-1, a regulatory molecule of the Wnt pathway, which led to impaired local bone formation. In addition, blockade of DKK-1 interfered with local bone resorption by reducing OCs numbers in the joints of animal models with inflammatory arthritis (45, 63, 64). A recent study of collagen-induced arthritis (CIA) in rats showed that TNF inhibition did not significantly affect RANKL, TNF-α, and OPG mRNA expression, which are linked to osteoclastogenesis, while it promoted bone formation via suppression of DKK-1 expression. This was confirmed by histomorphometric analysis, which demonstrated a significant increase in bone formation parameters while the changes in bone destruction parameters were not significant (63). Clinical trials in human RA confirmed the association of TNF with arthritic bone destruction as observed in several human TNF-α transgenic mouse models.

However, this observation was not clear in TNF-deficient mouse models, due to insignificant reduction in the clinical parameters of CIA and absence of major histological and morphological abnormalities in the joints. Moreover, some mice exhibited severe disease in the absence of TNF, suggesting the contribution of other cytokines such as IL-1 (65–68).

## TNF-α and Inflammatory Joint Diseases

Tumor necrosis factor-α has been associated with an increasing number of chronic inflammatory diseases. Among these diseases, the two most common inflammatory joint diseases RA and AS will be discussed. RA causes massive destruction of juxta-articular and systemic bone while AS leads to the concomitant bone destruction and ectopic bone formation (especially at the vertebral site) because of inflammation of tendon/ligament insertions. Thus, patients with AS experience stiffness of the spine known as ankylosis, and functional disability (69–71). Inhibition of TNF-α has substantially advanced the treatment of these two inflammatory diseases.

### Rheumatoid arthritis

Rheumatoid arthritis is a chronic disease characterized by joint inflammation leading to destruction of cartilage and bone (71). In RA, focal bone loss is due to an excessive bone resorption by OCs (72). Moreover, bone formation by OBs is greatly impaired at the erosion site in RA (73). TNF-α plays an important role in the bone destruction observed in RA patients. TNF inhibitors delay appearance of bone erosion in RA patients with no progression of bone destruction in responders and possibly to some extend, in non-responders (9, 74–76). In a murine CIA model, neutralization of TNF-α reduced joint pathology, especially when the treatment was started early after the onset of disease (77). Moreover, simultaneous inhibition of TNF-α and IL-17A further improved protection against joint damage even at the late stages of CIA (78). Mechanistic models of human TNF transgenic mice confirmed that ongoing TNF-driven inflammation suppresses new bone formation by Wnt signaling through up-regulation of DKK-1, and then the release of this inhibition allows new bone formation (45). Furthermore, several murine autoimmune arthritis models provided evidence that TNF deficiency reduces disease incidence (79–81).

The pro-inflammatory influence of TNF-α in RA acts at several levels. It induces other pro-inflammatory cytokines such as IL-1 and IL-17 and regulates osteoclastogenesis via several mechanisms. TNF-α accelerates cartilage destruction through inducing the production of matrix metalloproteinases (MMPs) and aggrecanases (ADAMTSs), which are enzymes that degrade components of the cartilage extracellular matrix. As an example, in human RA patients and in the human TNF-α transgenic mouse model, TNF-α was shown to regulate MMP3 and 13 by the small ubiquitin-like modifier-1 (SUMO-1) through NF-κB, which led to bone destruction (82). In addition, TNF-α stimulates OCs maturation both directly and indirectly by inducing RANKL on synovial fibroblasts, the synovial lining cells, and T-cells in the synovium (52, 83–85). This increased OCs maturation, and in turn, osteoclastic bone resorption. Moreover, TNF-α possibly promotes OCs activity via enhancing oxidative stress. The production of reactive oxygen species (ROS) by OCs helps accelerate the destruction of calcified tissue (86). This was confirmed in RA patients using an anti-TNF-α antibody therapy. In these patients, reduced levels of oxidative stress markers were associated with the control of joint and bone damage (87). Thus, TNF-α in RA has a destructive function as net effect.

### Ankylosing spondylitis

Ankylosing spondylitis is the second most common form of chronic inflammatory arthritis. The unique hallmark of the disease is pathologic new bone formation (88). AS and related spondyloarthritis are characterized by a paradoxical and simultaneous bone destruction and formation occurring in proximal anatomical sites. The strongest evidence for a key role of TNF-α in AS pathophysiology comes from *in vivo* inhibition of TNF in AS patients. TNF blockers reduce signs and symptoms of AS, which translates into better physical function and quality of life. However, little or no effect on structural remodeling is achieved (89, 90).

In AS, relevant human samples are not readily available even so TNF expression has been shown at the site of sacroiliitis. Thus, most observations are only made in rodent models. Recently, mice that specifically overexpress the transmembrane but not the soluble form of TNF-α, so called tmTNF transgenic mice, developed clinically spinal abnormalities similar to those in AS (91). These mice developed pronounced local inflammatory infiltration of the axial and peripheral joints (88). In sharp contrast with the other TNF overexpression models, these mice do not develop systemic inflammation, yet display pronounced axial and peripheral new bone formation leading to ankylosis (92). This model strongly argues for a role of tmTNF in the pathogenesis of AS but the exact molecular mechanisms still need to be elucidated. AS patients are particularly prone to develop syndesmophytes, a bony growth originating inside a ligament, and enthesitis, an inflammation at the sites where tendons or ligaments insert to bone. Therefore, in these settings, OCs may not be in direct contact with tendon-derived OBs and TNF-α may exhibit an osteogenic effect there. This is different from the effect found in whole bone where massive bone loss is seen. Thus in AS, even with TNF-α inhibition, ectopic bone formation is still observed, meaning that additional factors are governing this effect.

### Osteoporosis

Osteoporosis is characterized by a decrease in bone mass and density leading to bone weakness and prone to fracture. In RA patients, several studies have shown an increased incidence of osteoporosis. This could be due to the highly systemic inflammatory environment observed in these patients and the use of glucocorticoid treatment, which can affect bone remodeling (93, 94). Despite ectopic bone formation, AS patients show massive systemic osteoporosis (95). Among other cytokines, TNF-α seems to be an important player implicating in osteoporosis via TNFR1 (96). In RA patients treated with TNF-α inhibitors, control of bone loss was observed in parallel to that seen on arthritis levels (76, 97).

## Conclusion

Tumor necrosis factor-α has an important role in the regulation of bone homeostasis via activation of complex signaling pathways leading to gene transcription of several regulators of bone homeostasis. The classical view remains that TNF-α is an established inducer of osteoclastogenesis and inhibitor of osteoblastogenesis. To add further complexity, it was recently discovered that TNF-α also possessed osteogenic differentiation effects. Most of these paradoxical effects were only described in *in vitro* models and were concentration- and time-dependent, and cell-type specific. Despite the fact that TNF-α levels are high in both RA and AS, the bone dynamics are different. In RA, the dynamics are shifted toward bone destruction, combined with inhibition of specific osteogenic genes. In AS, systemic bone destruction is accompanied by ectopic bone formation at sites where OCs and OBs are disconnected. Future research should focus on the delineation of the molecular pathways used by this mediator and understand the conditions affecting and the purpose of this dual role in bone remodeling.

## Conflict of Interest Statement

The authors declare that the research was conducted in the absence of any commercial or financial relationships that could be construed as a potential conflict of interest.
